# Signal Pathways in Cancer

**DOI:** 10.3390/ijms24098260

**Published:** 2023-05-05

**Authors:** Guanbin Song

**Affiliations:** Key Laboratory of Biorheological Science and Technology, Ministry of Education, College of Bioengineering, Chongqing University, Chongqing 400030, China; song@cqu.edu.cn; Tel.: +86-23-65102507

Cancer is a major health-threatening disease and is the second leading cause of death worldwide; the prevention and treatment of cancer remains one of the most challenging problems clinically [1]. Therefore, exploring new effective ways to increase early diagnosis and improve the treatment of cancer in are great demand all over the world.

The occurrence and development of cancer is a slow and complicated process, which is closely related to the bidirectional interactions between cancer cells and their environment, forming the tumor microenvironment (TME). The TME is a complex mixture composed of cancer cells, stromal cells, immune cells, stem cells, tumor-associated fibroblasts (TAF), non-cellular components within extracellular matrix (ECM), etc., and plays a crucial role in carcinogenesis, growth, angiogenesis, invasion, metastasis and the therapeutic efficacy of cancer. There are various factors affecting cancer cells in the TME, including chemical factors, mechanical factors, biological factors and their coupling effects. Crosstalk between tumor cells and these factors is critical and contributes to tumor survival, progression and metastasis, which involves complex signal transmission/transduction processes. Despite the fact that many efforts have been made to elucidate crosstalk between cancer cells and the tumor microenvironment, the signal pathways in cancer remain far from clarified.

Based on this, a Special Issue entitled “Signal Pathways in Cancer” of the *International Journal of Molecular Sciences* was proposed in early 2022. In this Special Issue, a total of six contributions (three original articles and three reviews) are included, providing new information about the interaction between cancer cells and the TME and facilitating a better understanding of signal pathways in cancer.

The invasion and metastasis of cancer cells is one of the hallmarks of cancer [2]; however, active migration of cancer cells is a prerequisite for cancer cell invasion and metastasis, which is highly dependent on dynamic changes in the cytoskeleton. Plectin is a well-established regulator of cytoskeletal homeostasis and is involved in various physiological and pathological processes of many cell types. In recent years, plectin has been found to be upregulated in various cancer types, but the link between plectin expression, cancer cell invasion and metastasis is not fully understood. Xu et al. [3] investigated the role of plectin expression in the migration of hepatocellular carcinoma (HCC) cells and its relative molecular mechanism. The authors found that plectin expression is significantly increased in HCC tissue and cells compared to normal liver tissue and cells. They confirmed that plectin downregulation inhibits migration and suppresses the epithelial mesenchymal transformation (EMT) of HCC cells and revealed the critical roles of ERK1/2 signaling in this process. These findings provide a novel prognostic biomarker and potential therapeutic target for HCC in clinical settings.

ADAMTS16 is a member of the ADAMTS (a disintegrin and metalloproteinase with thrombospondin motifs) protein family and has been reported to be involved in the pathogenesis of solid cancers. The role of ADAMTS16 in gastric cancer was explored by Li et al. [4] by analyzing its effects on cell proliferation, migration and invasion. They observed the upregulation of ADAMTS16 in gastric cancer cell lines and that ADAMTS16 upregulates the IFI27 protein through the NF-κB pathway. Furthermore, the authors demonstrated that ADAMTS16 promotes gastric cancer cell migration, invasion and proliferation by targeting IFI27 through the NF-κB pathway. Taken together, these obtained results suggest that ADAMTS16 could be a potential prognostic biomarker of gastric cancer.

Increasing evidence has suggested a link between the nucleoli and cancer. The nucleolar stress response regulates the balance between proper protein synthesis and cell cycle progression and may be involved in the regulation of cancer sensitivity to certain drugs. However, the relationship between the nucleolar stress response and topoisomerase inhibitor sensitivity in human cancers is unknown. To address this question, using rhabdomyosarcoma and rhabdoid tumor cell lines, Ishihara et al. [5] examined the effect of the ribosomal protein L11 (RPL11)-mediated nucleolar stress response on the sensitivity to topoisomerase inhibitors. In this interesting study, they showed that after treatment with topoisomerase inhibitors, cell viability significantly increased upon RPL11 knockdown. Moreover, activation of the p53 pathway was decreased upon RPL11 depletion after treatment with topoisomerase inhibitors. These results provide evidence that the RPL11-mediated nucleolar stress response affects the sensitivity of cancer cells to topoisomerase inhibitors by regulating the p53 signaling pathway.

In recent years, signal pathways in cancer have been receiving growing attention due to their key roles in carcinogenesis and progression. The TME is crucial in neoplastic transformation, tumor growth, invasion, metastasis and protection of cancer cells from the host immune response, which involves a complex interplay between signaling pathways and TME components. Based on the reported evidence, Hamouda et al. [6] focused on colorectal cancer (CRC) and extensively reviewed the signaling pathways linked to CRC and their implication in the development or inhibition of tumor initiation and progression. In addition, they also summarized the major components of the TME, discussed the complexity of their cell functions and highlighted the double-edged sword effect of TME cells, as they play both pro- and anti-tumor roles. Their review not only provides a better understanding of the complexity and the dual function of these different actors in contributing to the failure of CRC control, but also provides new opportunities for the development of personalized and efficient therapies for CRC.

It is well known that cancer cells must evade detection and destruction by the immune system for successful metastasis. A large number of reports have implicated Hedgehog-GLI (HH) signaling in suppressing the immune system and promoting an immunosuppressive TME, which has led to a review on emerging roles of HH signaling in cancer immunity by Giammona et al. [7]. In this work, the authors very comprehensively described the effects of HH signaling on cellular components of the adaptive and innate immune systems. They also presented recent discoveries elucidating how the function of HH is engaged by cancer cells and discussed the link between the HH pathway and immune checkpoint inhibitors. Furthermore, the future prospects of therapeutic options combining the HH pathway and immune checkpoint inhibitors have been outlined. A deeper understanding of the effect of HH pathway activation and inhibition on the immune response is crucial for devising safe and effective therapies combining HH and immune checkpoint inhibitors.

Besides signal pathways in solid tumors, this Special Issue also included signaling in liquid tumors. Waldenström macroglobulinemia (WM) is an indolent malignant B cell lymphoma, with no standard therapy for effective treatment until now. The bone marrow microenvironment and cytokines both play pivotal roles in WM tumor progression. The mechanisms of disease progression and the current proposed therapies of WM are the subject of an extensive review by Boutilier et al. [8]. They gave a great overview of the available literature and summarized the current knowledge regarding the progression of and therapeutic strategies for WM. Their comprehensive review provides a better understanding of the role of the bone marrow microenvironment in the pathogenesis of WM and can help design improved therapeutic strategies for this disease in future clinical trials.

Cancer is a life-threatening disease and has been a major global public health problem for a long time. Research on the prevention, diagnosis and treatment of cancers has been booming for decades. It is acknowledged that the occurrence and progression of cancer is a multi-step, complicated, dynamic process involving various signaling pathways. Thus, research on signal pathways in cancer has been growing in recent decades. In fact, signal pathways in cancer are intricate and complex. As mentioned above, there are various signals in the TME, such as chemical, physical and biological signals, and they are accompanied by signaling networks. The important roles of mechanical signals have been recognized in the occurrence and progression of cancer in recent decades; unfortunately, there are no papers addressing mechanical signals (e.g., mechanotransduction) in cancer in this Special Issue. On the other hand, a small subpopulation of stem cells have been found within the TME in recent years, which are referred to as cancer stem cells (CSCs). CSCs have been proven to be the root of the cause, occurrence, progression, chemoradioresistance, recurrence and metastasis of cancer. Targeting CSCs is a novel therapeutic strategy for cancer treatment. However, there are also no papers involving CSC signals or the crosstalk between CSCs and the TME in this Special Issue. If possible, we will lead a new Special Issue on these topics in the near future. We believe that, with significant effort, scientists can eventually open this Pandora’s box, opening up new avenues for studying and treating cancer.

## Data Availability

All data are contained within the article.
